# 546. COVID-19 Monoclonal Antibody Use at a Stand Alone Children’s Hospital

**DOI:** 10.1093/ofid/ofad500.615

**Published:** 2023-11-27

**Authors:** Grant T Stimes, Brittany Rodriguez, Amanda Gillispie, Tanya J Hilliard, Flor M Munoz, Lisa Forbes Satter

**Affiliations:** Texas Children's Hospital, Houston, Texas; Texas Children's Hospital, Houston, Texas; Texas Children's Hospital, Houston, Texas; Texas Children's Hospital, Houston, Texas; Baylor College of Medicine, Houston, TX; Baylor College of Medicine, Houston, TX

## Abstract

**Background:**

Texas Children’s Hospital (TCH) implemented an outpatient clinic for eligible patients to receive infusions when COVID monoclonal antibodies (mAbs) were approved for use in non-hospitalized, high-risk patients with mild to moderate COVID-19. There are limited data evaluating the use of outpatient COVID mAbs in pediatric patients. We describe the clinical characteristics and outcomes of the patients treated with COVID mAbs at TCH.

**Methods:**

Patients that were referred to receive COVID mAbs from 12/1/2020 to 5/5/2022 were included. Information collected included demographics, comorbidities, refusal reason, adverse events, emergency center (EC) visits or admission within 14 days after final dose, and EC visit or admission within 14 days of referral if patients did not receive COVID mAbs. Chi-square was used to determine differences between the groups.

**Results:**

There were 262 patients referred during the study period. The median age of all referred patients was 15.8 years (IQR 14-17.4 years). Majority of referrals were for treatment of COVID-19 infection rather than post-exposure prophylaxis (92.8% vs 7.2%). Of the 262 referrals, 134 patients received COVID mAbs (51.2%) while 128 patients were not treated. Majority of the patients received casirivimab-imdevimab (73.9%). The comorbidities of all patients are shown in Figure 1. The most common reasons for patients to not receive COVID mAbs were no drug available or not listed, and the full list of reasons are listed in Figure 2. The median time from reported symptom onset to infusion was 4 days (IQR 2-6 days). Of the 134 infused patients, 8 patients (6%) visited the EC within 14 days from infusion, and 6 (4.5%) were admitted while 4 patients (3.1%) that did not receive an antibody visited the EC resulting in 3 admissions (2.3%) (EC visits p=0.27; admissions p=0.34). There were 11 patients (8.2%) that experienced adverse events from their infusion, which led to 5 of the 8 EC visits. Comorbidities were similar across the infused and non-infused groups (p=0.16).

**Figure:**
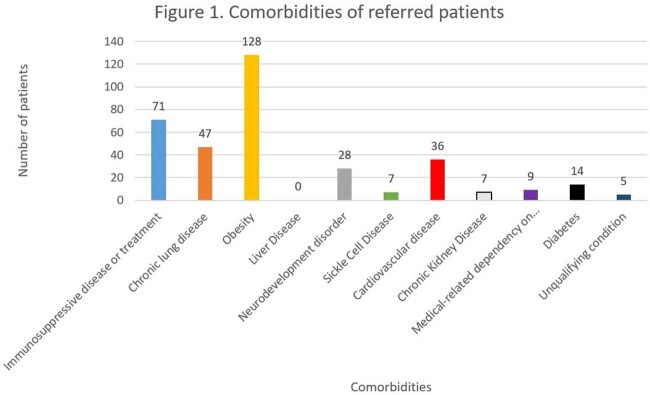

Number of high-risk conditions represented by all referred, high-risk patients

**Figure:**
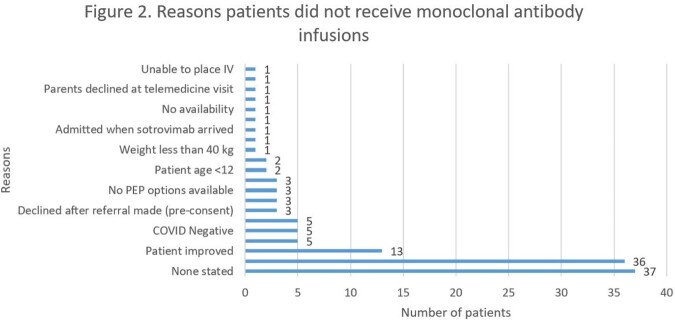

Number of patients for each reason for not receiving a COVID-19 antibody infusion

**Conclusion:**

COVID mAb treatments were well tolerated among pediatric patients. Majority of patients in both groups did not require EC visit or hospitalization. More data are needed to determine the clinical efficacy of mAbs patients.

**Disclosures:**

**Flor M. Munoz, MD, MSc**, CDC respiratory virus surveillance: Grant/Research Support|Gilead: Grant/Research Support|Moderna, sanofi, aztra zeneca, Merck, GSK: Advisor/Consultant|NIH: DSMB|NIH COVID-19 vaccines in pregnancy: Grant/Research Support|Pfizer Pediatric COVID-19 vaccines: Grant/Research Support|Pfizer, Dynavax, Monderna, Meissa, NIH: DSMB **Lisa Forbes Satter, MD**, ADMA: Advisor/Consultant|CsL Behring: Advisor/Consultant|Grifols: Advisor/Consultant|incyte: Advisor/Consultant|Pharming: Advisor/Consultant|Takeda: Advisor/Consultant

